# Biomarkers of Diabetic Macular Edema on Optical Coherence Tomography After Cataract Surgery

**DOI:** 10.1177/24741264261423317

**Published:** 2026-03-19

**Authors:** Tuan Tran, Jonathon Goh, Sophie Rogers, Salmaan Qureshi, Lyndell L. Lim

**Affiliations:** 1 Centre for Eye Research Australia, East Melbourne, VIC, Australia; 2 The Royal Victorian Eye and Ear Hospital, East Melbourne, VIC, Australia; 3 Griffith University, School of Medicine and Dentistry, Sunshine Coast Health Institute, Birtinya, QLD, Australia; 4 Department of Ophthalmology, Sunshine Coast University Hospital, Birtinya, QLD, Australia; 5 University of Melbourne, Parkville, VIC, Australia

**Keywords:** diabetic macular edema, diabetic retinopathy, cataract, optical coherence tomography, biomarkers

## Abstract

**Purpose:** To evaluate how 6 features of diabetic macular edema (DME), including intraretinal cyst size, ellipsoid zone (EZ) integrity, disorganization of the retinal inner layers (DRIL), hyperreflective foci, subretinal fluid, and foveal contour, evolve on optical coherence tomography (OCT) after cataract surgery with intraoperative and ongoing postoperative treatment with intravitreal (IVT) bevacizumab or triamcinolone. Secondary objectives included correlating these OCT features with best-corrected visual acuity (BCVA). **Methods:** In the Diabetic Macular Edema at the time of Cataract Surgery trial, patients with cataracts and center-involving DME were randomized at the time of cataract surgery to IVT triamcinolone or bevacizumab. Both treatment groups were included in this post-hoc analysis. Baseline preoperative macular OCT scans were obtained and at 1, 3, and 6 months postoperatively. Imaging features were assessed and graded as per the TCED-HFV protocol. An overall grading score of OCT structural changes was developed to explore the predictability of BCVA. The associations between OCT features of DME and BCVA were assessed with the Wilcoxon rank-sum test. **Results:** Forty-five eyes (42 patients) were included in the analysis. OCT features remained stable over the follow-up period. BCVA was correlated with increased intraretinal cyst size, the presence of DRIL, EZ disruption or loss, and TCED-HFV score. Baseline TCED-HFV scores predicted visual outcomes and remained stable. **Conclusions:** Imaging features of DME remained stable with postoperative treatment. Visual outcomes correlated with OCT features and staging, emphasizing the predictive role of preoperative imaging biomarkers.

## Introduction

Visually significant cataracts and coexistent diabetic macular edema (DME) is a common clinical scenario affecting many patients. Currently, it is estimated that 1.3 million Australians suffer from diabetes, with 50 000 having clinically significant macular edema. The odds ratio of a patient with diabetes having a visually significant cataract is 1.29 to 1.97 compared with a patient without diabetes.^1–3^

The inflammatory state produced during cataract surgery is known to exacerbate diabetic retinopathy and DME, with historically poor visual outcomes.^4–6^ Recent advances in the management of DME, namely intravitreal (IVT) corticosteroids or antivascular endothelial growth factor (anti-VEGF) agents, have greatly improved outcomes in both nonoperative and operative settings.^7–9^

The Diabetic Macular Edema at the time of Cataract Surgery trial (DiMECAT) was one of the first prospective, randomized clinical trials providing evidence that adjuvant IVT steroids during and after cataract surgery had a longer-lasting effect and reduced postoperative central macular thickness (CMT) more effectively compared with IVT anti-VEGF agents. In the group receiving IVT triamcinolone, fewer injections were required in the postoperative period, and the CMT was thinner compared with patients receiving IVT bevacizumab.^8^ However, the better CMT outcomes did not translate into greater gains in visual acuity (VA), a finding that was supported by a recent meta-analysis comparing anti-VEGF and dexamethasone implants in the setting of cataract surgery.^9^ This highlights the likelihood of other anatomic changes at the macula beyond CMT that may have a bearing on VA outcomes. Diabetic retinopathy and DME result in multiple structural changes identifiable by optical coherence tomography (OCT) imaging, with recognized biomarkers being associated with poor visual prognosis. These structural changes include disorganization of the retinal inner layers (DRIL), disruption of the ellipsoid zone/external limiting membrane (EZ/ELM), presence of hyperreflective foci, the presence of intraretinal and subretinal fluid (SRF), and changes to the CMT.^10^

Although it has been well documented that the CMT in patients with DME tends to increase after cataract surgery,^7,8,11^ the effect of cataract surgery on individual structural OCT changes, to our knowledge, has not previously been studied. Assessing how these microstructural changes evolve postoperatively could help predict visual outcomes after cataract surgery and would greatly assist in the management and counseling of patients facing the common scenario of coexistent DME and visually significant cataracts. Furthermore, how adjuvant IVT steroids or anti-VEGF agents may affect these individual structural changes postoperatively is also unknown.

In the current study, a post-hoc analysis of the DiMECAT study data was performed to investigate how the perioperative use of either IVT triamcinolone or bevacizumab influences various structural OCT changes of DME postoperatively. We also assessed whether these structural OCT changes may relate postoperatively to best-corrected visual acuity (BCVA).

## Methods

This post-hoc analysis assessed the preoperative and postoperative macula OCT B-scan images of participants in the DiMECAT trial, the methodology of which has previously been described.^12^

Patients with visually significant cataract and center-involving DME, either current or treated within the previous 24 months, were recruited from the Royal Victorian Eye and Ear Hospital. Exclusion criteria included other causes of macular edema apart from diabetes, patients who had received macular laser within 12 weeks, IVT triamcinolone within 12 weeks, or IVT bevacizumab within 10 weeks. Eligible patients were block randomized in a 1:1 ratio at the time of cataract surgery to receive either IVT triamcinolone or bevacizumab. Patients were reviewed at baseline (up to 2 weeks preoperatively), 1 week postoperatively, then monthly for 6 months. At each review appointment, assessment included logMAR BCVA testing with Early Treatment Diabetic Retinopathy Study (ETDRS) charts, intraocular pressure, and slitlamp examinations of the anterior and posterior segments. Fast macular thickness scans and 6 mm crosshair scans were obtained using Spectralis OCT (Heidelberg Engineering). The pattern size of the OCT scans was 20.0 degrees × 20.0 degrees (6.2 mm × 6.2 mm) with a distance of 252 µm between B-scans for a total of 25 B-scans. Fundus color photography was performed at baseline and at 6-month visits (Topcon TRC-50EX, Topcon Corporation).

All patients underwent standard phacoemulsification surgery and insertion of an AcrySof SA60AT intraocular lens (Alcon) followed by IVT injection of the randomly assigned agent (triamcinolone or bevacizumab). Postoperatively, all participants were treated with the same topical treatment regimen of prednisolone acetate 1% (Allergan) and chloramphenicol 0.5% (Sigma Pharmaceuticals) 4 times a day, tapered over 4 weeks. IVT injections could be repeated if at least 77 days had passed from the patient’s last IVT triamcinolone treatment or at least 28 days from their last IVT bevacizumab treatment; and if CMT increased by 50 µm or more compared with the best recorded CMT in the study, or a decrease of 5 or more ETDRS letters compared with the best recorded BCVA. The study protocol adhered to the tenets of the Declaration of Helsinki, and ethical approval was obtained from the Human Research Ethics Committee of the Royal Victorian Eye and Ear Hospital. Written informed consent was obtained from all patients before participation.

For the purposes of this post-hoc analysis, 6-month follow-up data from the DiMECAT trial were used. Preoperative horizontal B-scans centered on the foveola, performed within 2 weeks of surgery, as well as 1-, 3-, and 6-month postoperative examinations were graded. Eyes must have had a gradeable B-scan at both the preoperative and 6-month examination to be included in the study. Each scan was graded according to the European School for Advanced Studies in Ophthalmology classification, also known as the TCED-HFV grading scheme (Table 1).^10^ This grading scheme uses 7 qualitative and quantitative OCT indicators to describe the progression of DME and enables the severity of DME to be standardized.^13–15^ The TCED-HFV grading scheme was developed to explore the associations between structural macula changes of DME seen on OCT and concurrent BCVA and postoperative visual prognosis (Table 2).

**Table 1.** Baseline Characteristics of Eyes Excluded and Included in the DiMECAT Study That Were Included in the Current Post-Hoc Analysis.

Baseline Characteristics of Patients	Excluded (n = 16)	Included (n = 42)	*P* Value
Mean age of eye at surgery, (95% CI)	65.4 (60.0-70.9)	67.8 (65.3-70.3)	.42
Female sex, n (%)	5 (31)	14 (33)	1.0
Diabetes type, n (%)			.19
T1 IDDM	0	1 (2)	
T2 NIDDM	9 (56)	13 (31)	
T2 IDDM	7 (44)	28 (67)	
Median HbA_1c_, %, IQR	7.6 (7.1, 9.2)	7.4 (6.9, 8.1)	.38
Diabetic retinopathy at baseline, n (%)			.69
Mild NPDR	3 (19)	4 (9)	
Moderate NPDR	6 (38)	18 (40)	
Severe NPDR	1 (6)	7 (16)	
PRP only (inactive)	6 (38)	14 (31)	
Treated PDR (active)	0	2 (4)	
Baseline Characteristics of Eyes	Excluded (n = 16)	Included (n = 45)	
Median CMT (µm), IQR	344 (274, 469)	305 (284, 430)	.58
CMT >300 µm, n (%)	10 (63)	27 (60)	1.0
DME dry at surgery, n (%)	7 (44)	25 (56)	.56
Mean IOP, mm Hg, (95% CI)	14.8 (12.7-16.9)	14.8 (14.0-15.6)	.97
Mean VA (ETDRS letters), (95% CI)	46 (37-55)	54 (50-59)	.094
Past focal laser treatment, n (%)	10 (63)	24 (53)	.572
DiMECAT treatment group, n (%)			1.0
Triamcinolone	9 (56)	24 (53)	
Bevacizumab	7 (43)	21 (47)	

Abbreviations: CMT, central macular thickness; DiMECAT, Diabetic Macular Edema at the time of Cataract Surgery; DME, diabetic macular edema; ETDRS, Early Treatment Diabetic Retinopathy Study; HbA_1c_, glycosylated hemoglobin; IDDM, insulin-dependent diabetes mellitus; IOP, intraocular pressure; IQR, interquartile range; NIDDM, noninsulin-dependent diabetes mellitus; NPDR, nonproliferative diabetic retinopathy; PDR, proliferative diabetic retinopathy; PRP, panretinal photocoagulation; VA, visual acuity.

**Table 2.** Study Eyes With Abnormal Preoperative Baseline OCT Features and Eyes That Improved at 6 Months (Compared With No Change or Worsening), Overall and by DiMECAT Treatment Group.

OCT Feature	Overall		TA		BVB		*P* Value^a^
	Eyes With Feature Present at Baseline, n (%)	Rate of Improvement at 6 Months per 100 Eyes, (95% CI)	Eyes With Feature Present at Baseline, n (%)	Rate of Improvement at 6 Months per 100 Eyes, (95% CI)	Eyes With Feature Present at Baseline, n (%)	Rate of Improvement at 6 Months per 100 Eyes, (95% CI)	
Central subretinal thickness (>10%)	16 (36%)	50 (28-72)	10	60 (31-83)	6	33 (9-70)	.302
Intraretinal cyst size (mild, moderate, or severe)	32 (71%)	34 (20-52)	17	47 (26-69)	15	20 (6-46)	.108
EZ/ELM status (disrupted or absent)	27 (60%)	15 (5-33)	13	8 (0-35)^b^	14	21 (7-48)	.315
Vitreomacular relationship (ERM or VMT)	14 (31%)	14 (3-41)	6	17 (1-58)	8	13 (0-49)	.826
Pattern of DME (DME or mixed pattern)	36 (80%)	14 (6-29)	18	22 (8-46)	18	6 (0-28)^b^	.148
Foveal contour (greater than surrounding macula)	17 (38%)	53 (31-74)	9	67 (35-88)	8	38 (13-70)	.229
Disorganization of retinal layers (present)	20 (44%)	10 (2-31)	12	8 (0-38)^b^	8	13 (0-49)	.761
Hyperreflective foci (>30+)	2 (4%)	50 (9-91)	1	0 (0-83)^b^	1	100 (17-100)^c^	NA
Subretinal fluid (present)	3 (7%)	100 (38-100)^c^	1	100 (17-100)^c^	2	100 (29-100)^c^	NA
Stage of DME (Early, advanced, severe, or atrophic)	35 (80%)	14 (6-30)	18	17 (5-40)	17	12 (2-36)	.679

Abbreviations: DiMECAT, Diabetic Macular Edema at the time of Cataract Surgery; DME, diabetic macular edema; ELM, external limiting membrane; ERM, epiretinal membrane; EZ, ellipsoid zone; NA, not applicable; VMT, vitreomacular traction.

a *P* value relates to the 2-sample test of proportions between the treatment arms of DiMECAT study.

b CI clipped at lower end and may not have true 95% coverage.

c CI clipped at upper end and may not have true 95% coverage.

Due to the paucity of literature regarding how the foveal contour relates to visual function in the setting of DME and cataract surgery, the foveal contour was graded in a similar fashion as to how it is graded with regards to vitreomacular traction (Table 1).^16^ Each OCT B-scan was independently graded by 2 masked retina specialists (J.G., T.T.). Any disagreements were adjudicated by joint review of the scan in question. Intergrader reliability was quantified with Cohen’s unweighted kappa.

Continuous variables were summarized as means (95% CIs) or medians (interquartile range) as appropriate to their distribution. Categorical variables were summarized as counts and percentages. Wilcoxon rank-sum test was used to assess the associations between OCT structural features and BCVA at each time point. Kendall’s tau rank correlation test was used to assess the relationships between the grading score and concurrent BCVA at each time point in addition to the preoperative grading score and the final 6-month BCVA. The incidence of OCT features by 6 months in patients with absence of the feature at the preoperative examination was estimated with time-to-event analysis, using all available grading examinations. Statistical analyses were performed with Stata (StataCorp LLC), with *P* < .05 considered significant.

## Results

Of the 61 eyes (58 patients) recruited into the DiMECAT study, 16 eyes (from 12 patients) were excluded due to incomplete data/images, leaving 45 eyes (42 patients) eligible for analysis, which assessed both the IVT triamcinolone and bevacizumab treatment groups together. No significant differences were found in baseline demographics between included and excluded patients (Table 1).

The patients’ mean age was 67.8 years (95% CI, 65.3-70.3), and the mean baseline BCVA was 54 ETDRS letters (95% CI, 50-59). The intergrader reliability ranged from 80.5% to 98.9% in agreement for each OCT parameter (Supplementary Table 1). This corresponds to moderate-to-strong agreement^17^ (unweighted kappa values ranging from 0.612-0.894), except for hyperreflective foci which, despite an agreement of 91.4%, had a minimal agreement (kappa = 0.345) that may be explained by the low prevalence of hyperreflective foci observed in the testing set and the kappa paradox.^18^

### OCT Measurements at Baseline

Table 2 shows the baseline imaging biomarkers of DME. At baseline, there were 16 eyes (36%) with central subfoveal thickness greater than 10%, 32 (71%) had intraretinal cyst (mild, moderate, or severe), 27 (60%) had disruption or absence of EZ/ELM, 20 (44%) had DRIL, 6 (13%) had an epiretinal membrane (ERM), 36 (80%) had DME pattern or mixed pattern of edema, 17 (38%) had foveal level at or greater than the surrounding macula, 2 eyes (4%) had more than 30 hyperreflective foci, and 3 eyes (7%) had SRF at baseline. Nine eyes (20%) had no DME at baseline as graded by TCED-HFV stage, 14 (31%) had early DME, 15 (33%) had advanced DME, 3 (7%) had severe DME, and 3 (7%) had atrophic DME. No statistically significant difference was found in any of the assessed OCT structural features between baseline and 6 months when the whole cohort was assessed together, nor was any statistically significant difference found in the prevalence of any of the graded OCT structural features between the 2 groups at all time points (Table 2).

### OCT Measurements: Baseline vs 6 Months

Table 3 demonstrates the changes in grade of each OCT feature from baseline to 6 months. There were 29 eyes with central subfoveal thickness of 10% or less at baseline. At 6 months, 24 of these eyes continued to have central subfoveal thickness of 10% or less, 4 (all from the IVT bevacizumab group) developed central subfoveal thickness of 10% to 29%, and 1 (IVT bevacizumab group) developed central subfoveal thickness of 30% or more. Of the 12 eyes that had central subfoveal thickness of 30% or more at baseline, 5 eyes (4 in the IVT triamcinolone group, 1 in the IVT bevacizumab group) improved to 10% or less, 3 (2 in the IVT triamcinolone group, 1 in the IVT bevacizumab group) improved to 10% to 29%, and 4 (2 in the IVT triamcinolone group, 2 in the IVT bevacizumab group) continued to have central subfoveal thickness of 30% or more at 6 months.

**Table 3.** Changes in OCT Features From Baseline to 6 Months.

Central Subfoveal Thickness					
At 6 Months					
At baseline	≤10%	10%-29%	≥30%	Total	
≤10%	24	4	1	29	
+10%-29%	0	2	2	4	
≥30%	5	3	4	12	
Total	29	9	7	45	
Intraretinal Cyst Size					
At 6 Months					
At baseline	Absent	Mild	Moderate	Severe	Total
Absent	10	3	0	0	13
Mild	4	13	4	1	22
Moderate	1	3	1	1	6
Severe	1	1	1	1	4
Total	16	20	6	3	45
Ellipsoid Zone and/or External Limiting Membrane					
At 6 Months					
At baseline	Intact	Disrupted	Absent	Total	
Intact	17	1	0	18	
Disrupted	3	18	1	22	
Absent	0	1	4	5	
Total	20	20	5	45	
Disorganization of Retinal Inner Layer					
At 6 Months					
At baseline	None	Present	Total		
None	18	7	25		
Present	2	18	20		
Total	20	25	45		
Vitreomacular Relationship					
At 6 Months					
At baseline	Absent	Incomplete	VMT	ERM	Total
Absent	24	3	0	4	31
Incomplete	1	6	1	0	8
ERM	1	0	0	5	6
Total	26	9	1	9	45
Pattern of DME					
At 6 Months					
At baseline	No edema	DME	Mixed	Total	
No edema	9	0	0	9	
DME	4	27	4	35	
Mixed	1	0	0	1	
Total	14	27	4	45	
Foveal Contour					
At 6 Months					
At baseline	Normal	F=Surround	F>macular	Total	
Normal	23	2	3	28	
F=Surround	5	3	0	8	
F>Macula	2	2	5	9	
Total	30	7	8	45	
Hyperreflective Foci					
At 6 Months					
At baseline	<30	≥30	Total		
<30	42	1	43		
≥30	1	1	2		
Total	43	2	45		
Subfoveal Fluid					
At 6 Months					
At baseline	None	Present			
None	42	0	42		
Present	3	0	3		
Total	45	0	45		

Abbreviations: DME, diabetic macular edema; ERM, epiretinal membrane; VMT, vitreomacular traction.

There were 13 eyes with absent intraretinal cyst size at baseline. Only 3 (1 in the IVT triamcinolone group, 2 in the IVT bevacizumab group) of these eyes worsened to mild intraretinal cyst size at 6 months, and the remaining 10 (6 in the IVT triamcinolone group, 4 in the IVT bevacizumab group) were stable. Of the 18 eyes that had intact EZ/ELM at baseline, 17 remained intact at 6 months, and only 1 (IVT triamcinolone) worsened to have disrupted EZ/ELM. Of the remaining 27 eyes, 5 (3 in the IVT triamcinolone group, 2 in the IVT bevacizumab group) had absent EZ/ELM, and 22 (10 in the IVT triamcinolone group, 12 in the IVT bevacizumab group) had disrupted EZ/ELM at baseline. At 6 months, none of the 5 eyes initially graded as absent EZ/ELM had recovery of the EZ/ELM. Of the 22 eyes with EZ/ELM disruption at baseline, only 3 (1 in the IVT triamcinolone group, 2 in the IVT bevacizumab group) had recovered to an intact EZ/ELM at 6 months. The incidence of developing disrupted or absent EZ/ELM by 6 months was 3.2 per 100 patients in eyes with intact EZ/ELM at baseline.

There were 25 eyes (12 in the IVT triamcinolone group, 13 in the IVT bevacizumab group) without DRIL at baseline, of which 7 (3 in the IVT triamcinolone group, 4 in the IVT bevacizumab group) developed DRIL by 6 months. In contrast, 20 eyes (12 in the IVT triamcinolone group, 8 in the IVT bevacizumab group) had DRIL at baseline, 18 had persistent DRIL, and only 2 (1 in the IVT triamcinolone group, 1 in the IVT bevacizumab group) had resolution of DRIL at 6 months. The incidence of new DRIL developing by 6 months in patients without DRIL at baseline was 6.6 per 100 patients. At baseline, 24 eyes had absent vitreomacular disorders; 3 of 24 eyes had incomplete posterior vitreous detachment at 6 months, and 4 eyes had developed ERM. There were 43 eyes with less than 30 hyperreflective foci at baseline, and only 1 (IVT triamcinolone group) of these eyes developed more than 30 hyperreflective foci by 6 months. Two eyes (1 in the IVT triamcinolone group, 1 in the IVT bevacizumab group) had 30 or more hyperreflective foci at baseline, 1 (IVT bevacizumab group) of which improved to less than 30 by 6 months. The incidence of 30 or more hyperreflective foci by 6 months in those with less than 30 at baseline was only 1.2 per 100 patients. At 6 months, 42 eyes with no SRF at baseline remained without SRF, while 3 eyes with SRF at baseline had resolution of SRF.

Of the 9 eyes (6 in the IVT triamcinolone group, 3 in the IVT bevacizumab group) with TCED-HFV staging of no DME at baseline, 3 (2 in the IVT triamcinolone group, 1 in the IVT bevacizumab group) worsened to develop early DME at 6 months. Of the 14 eyes with early DME at baseline, 3 (2 in the IVT triamcinolone group, 1 in the IVT bevacizumab group) improved to no DME, 8 (4 in the IVT triamcinolone group, 4 in the IVT bevacizumab group) remained stable, and 3 (1 in the IVT triamcinolone group, 2 in the IVT bevacizumab group) worsened to advanced DME at 6 months. There were 15 eyes with advanced DME at baseline. One eye improved to early DME (1 in the IVT triamcinolone group), 12 (4 in the IVT triamcinolone group, 8 in the IVT bevacizumab group) remained stable with advanced DME, and 2 (both in the IVT triamcinolone group) worsened to atrophic DME at 6 months. Three eyes had severe DME at baseline; 1 (in the IVT triamcinolone group) remained severe, and 2 (1 from each group) progressed to atrophic DME at 6 months. Three eyes had atrophic DME at baseline, with only 1 eye (in the IVT bevacizumab group) improving to advanced DME at 6 months. There were 23 eyes with either no DME or early DME at baseline, of which only 3 eyes worsened (to advanced DME) at 6 months. Of the 21 eyes with baseline staging between advanced, severe, or atrophic DME, only 2 eyes (1 from each group) clinically improved from their baseline stage after 6 months (Table 4).

**Table 4.** Change in DME Stage From Baseline to 6 Months.

At 6 Months						
At Baseline	No DME	Early DME	Advanced DME	Severe DME	Atrophic DME	Total
No DME	6	3	0	0	0	9
Early DME	3	8	3	0	0	14
Advanced DME	0	1	12	0	2	15
Severe DME	0	0	0	1	2	3
Atrophic DME	0	0	1	0	2	3
Total	9	12	16	1	6	44

Abbreviation: DME, diabetic macular edema.

### OCT Structural Features and BCVA

**Figure 1.**
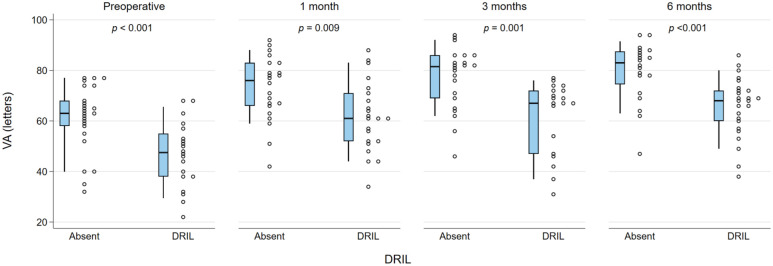
VA (letters) in the presence of DRIL compared with its absence (0) at each time point. The narrow-shaded boxes represent the interquartile range for VA at that timepoint, and whiskers indicate the 10th and 90th percentiles. The *P* values relate to Wilcoxon rank-sum test for differences between 2 groups. Abbreviations: DRIL, disorganization of the retinal inner layers; VA, visual acuity.

**Figure 2.**
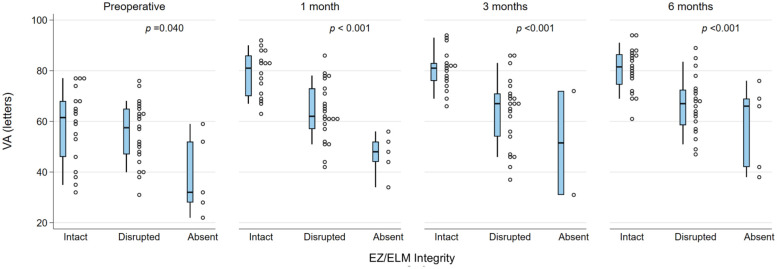
VA (letters) and EZ/ELM integrity grades at each time point. The narrow-shaded boxes represent the interquartile range for VA at that timepoint, and whiskers indicate the 10th and 90th percentiles. The *P* values relate to Kendall’s tau rank correlation test from trend across EZ/ELM integrity categories. Abbreviations: ELM, external limiting membrane; EZ, ellipsoid zone; VA, visual acuity.

**Figure 3.**
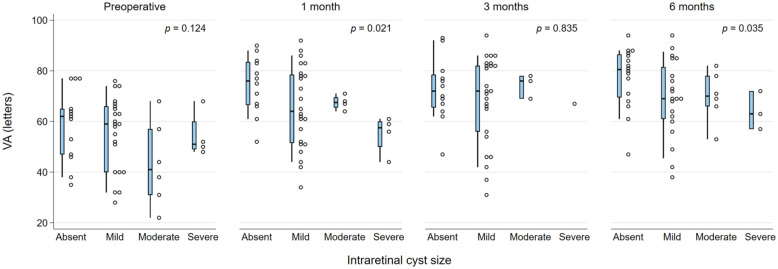
VA (letters) and intraretinal cyst size at each time point. The narrow-shaded boxes represent the interquartile range for VA at that timepoint, and whiskers indicate the 10th and 90th percentiles. The *P* values relate to Kendall’s tau rank correlation test for trend across intraretinal cyst severity categories. Abbreviation: VA, visual acuity.

**Figure 4.**
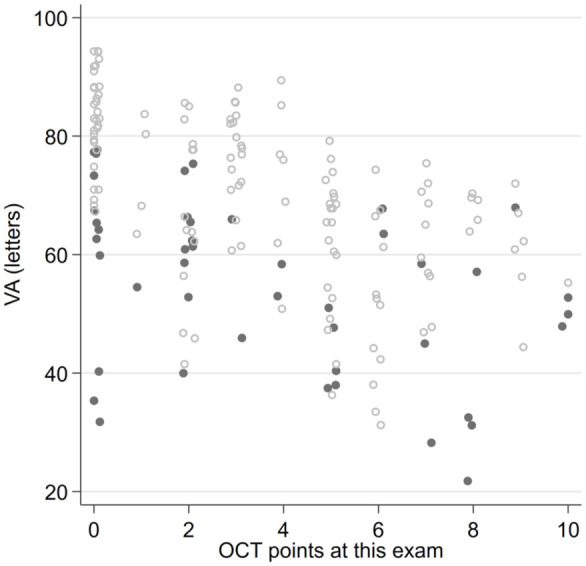
Relationship between OCT grading score and best-corrected VA at the baseline preoperative examination (shaded circles) compared with all postoperative timepoints combined (open circles). Abbreviations: OCT, optical coherence tomography; VA, visual acuity.

**Figure 5.**
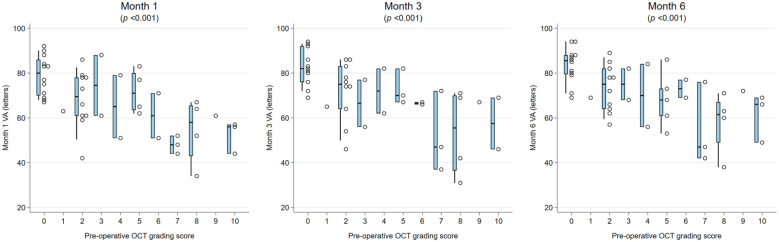
Relationship between baseline preoperative OCT grading score and postoperative VA. The narrow-shaded boxes represent the interquartile range for VA at that timepoint, and whiskers indicate the 10th and 90th percentiles. *P* values relate to Kendall’s tau rank correlation test. Abbreviations: OCT, optical coherence tomography; VA, visual acuity.

The presence of DRIL or EZ disruption or loss was consistently statistically significantly associated with worse BCVA at all postoperative time points (all *P* < .05). In addition, preoperative BCVA was worse in the presence of DRIL but did not reach statistical significance for DRIL (Figures 1 and 2). Foveal contour greater than the surrounding macula was significantly correlated with worse VA (*P* = .03) at 1 month but not at other time points. The intraretinal cyst grading was significantly associated with BCVA at 1 month (*P* = .015) and at 6 months (*P* = .02) (Figure 3). Hyperreflective foci, SRF, and foveal contour were not associated with BCVA at any time point, and there was no statistically significant difference in the presence of each OCT feature between the 2 groups. A higher OCT grading score was significantly associated with worse concurrent BCVA at all timepoints combined (*P* < .001) (Figure 4) and at each individual time point (*P* = .006 at baseline, *P* < .001 at months 1, 3, and 6). A higher preoperative grading score was also associated with a lower postoperative BCVA at all time points (*P* < .001) (Figure 5).

## Conclusions

DME manifests with a range of microstructural changes of the macula, and recent studies have demonstrated the importance of specific OCT imaging biomarkers.^19^ It is well known that patients with diabetes undergoing cataract surgery are at risk of developing or having worse DME^11^; however, there is a paucity of published data on how these OCT features beyond CMT behave in response to cataract surgery. To our knowledge, this post-hoc analysis is one of the first to assess individual OCT biomarkers in the treatment of DME (with either IVT triamcinolone or bevacizumab) at the time of cataract surgery. The main findings of this study demonstrate that the OCT stage of DME, along with many of the individual biomarkers, remained stable postoperatively with intensive treatment with either IVT triamcinolone or bevacizumab. Patients with a worse baseline TCED-HFV grading score, DRIL, or reduced EZ/ELM integrity are likely to retain these features, regardless of treatment, while those with better TCED-HFV scores may be able to maintain their mild disease status and good visual outcomes with ongoing IVT treatment.

The TCED-HFV grading scheme was designed to acknowledge the microstructural features of DME observed on OCT and employ a standardized approach to classifying and staging the disease severity.^10^ This grading scheme has been shown to be predictive of visual outcome with treatment for DME^10,13,14^; however, further studies are needed to independently validate this grading score and determine its practical use in the clinical setting. In our study, a more advanced TCED-HVF stage of DME was correlated with worse BCVA at all time points, and a worse preoperative stage was more likely to result in poorer postoperative visual outcomes.

Our cohort of eyes with a mild stage of DME (no or early DME TCED-HFV grading) remained stable or improved after cataract surgery; however, patients with a more severe baseline stage of DME (advanced, severe, or atrophic DME TCED-HFV grading) generally remained poor or even worsened at 6 months. This finding supports the common practice of optimizing the DME status before cataract surgery to lessen the damage associated with surgery and prevent progression to more severe DME postoperatively. Furthermore, these results may assist in the counselling of patients with DME regarding their visual prognosis before planned cataract surgery, as those found to have severe DME TCED-HFV grades can be given more guarded outcomes than those with milder scores.

The results from the current study also demonstrate that microstructural OCT features of DME remained largely stable in the postoperative setting at 6 months compared with baseline. In the DiMECAT study, eyes in the IVT triamcinolone group had a sustained reduction in CMT (−51.4 µm), whereas CMT increased mildly (+15.6 µm) in the IVT bevacizumab group.^8^ Reassuringly, many of the individual OCT biomarkers of DME remained stable for up to 6 months postoperatively in both groups. We found no differences in the overall prevalence and severity of these structural OCT features during the 6-month follow-up period compared with baseline, and there was no difference between the groups. The close monthly postoperative monitoring and prompt treatment with IVT triamcinolone or bevacizumab in this study is likely to have contributed to the stability of these microstructural OCT features. In a large UK-based study using real-world data from 4850 eyes of patients with diabetic retinopathy, but without DME, a significant increase was observed in the development of treatment-requiring DME after cataract surgery, with the peak incidence between 3 and 6 months.^11^ The authors suggested that increased monitoring and treatment is required within this time frame. Another possible reason the microstructural changes remained stable during our study’s 6-month follow-up is that some of these changes may take longer than 6 months to develop or to be identifiable by OCT.

In addition to the TCED-HFV grading score, the presence of DRIL and EZ/ELM disruption correlated with worse BCVA, supporting the importance of how these microstructural features affect BCVA in DME.^20–22^ Despite the strong association of these markers with poorer visual outcomes in patients with DME, how the status of DRIL and EZ/ELM evolves after cataract surgery has not been previously reported. In the current study, we have shown that these changes, if present preoperatively, are unlikely to resolve, even with intensive postoperative treatment with either IVT triamcinolone or bevacizumab. The integrity of the EZ/ELM tends to be maintained in eyes with intact EZ/ELM at baseline, with a low incidence of disruption by 6 months. Regarding DRIL, there was no overall difference in the incidence of preoperative DRIL compared with the status at 6 months. However, subgroup analysis found that a proportion of patients (7/25) without DRIL at baseline developed DRIL at 6 months, and most patients (18/20) with preexisting DRIL at baseline continued to have DRIL at 6 months. Thus, while most patients maintain a stable DRIL status, there is a subset of eyes that may, over time, develop DRIL after cataract surgery, which is associated with a worse visual outcome. As these microstructural features of DME are largely maintained after cataract surgery, identifying OCT biomarkers of DME preoperatively that are known to be strongly associated with BCVA, such as presence of DRIL and loss of EZ/ELM integrity, may be valuable in assessing and prognosticating patients’ postoperative visual potential.

Strengths of this study include the prospective collection of imaging data, the grading of all images by 2 masked graders with strong agreement across most variables graded, and the use of a standardized grading scheme. However, the relatively small sample size of both treatment arms is a limitation. Significant changes to some of these microscopic DME features may potentially only be elucidated with a larger population, but no trends were noted in our analysis. Follow-up was also limited to 6 months, which may be inadequate to identify and determine microstructural changes that take longer to develop or resolve. We also acknowledge that OCT imaging features may behave differently in response to treatment with the various anti-VEGF and corticosteroid agents available, apart from IVT triamcinolone and bevacizumab.

There are also other OCT-based DME grading schemes aside from the TCED-HCF,^23–25^ and while a strength of this system is its incorporation of multiple different characteristics beyond just CMT, SRF, and cysts, some limitations exist. For example, its grading of CMT in 3 discrete groups (0%-10% increase, 10%-30% increase, and ≥30% increase) rather than a continuous variable to measure severity. This may explain the difference between the initially reported CMT changes (increased in the IVT bevacizumab group and decreased in the IVT triamcinolone group) and that of the data in the current study (no overall change) appearing contradictory. Patients assigned the highest CMT grading (≥30%) have no upper limit. Likewise, TCED-HCF grades SRF as either absent or present, so SRF height is not measured as a continuous variable. Despite this, simplifying these OCT features into discrete variables may be less time consuming and be deemed more practical and appropriate to implement in the clinical setting. A further limitation with the grading scheme is that the measurement of certain OCT features may not reflect a true association with BCVA. For example, the atrophic stage of the TCED-HFV grading system may be classified by the presence of degenerative macrocysts or thick CMT.^10^ However, in diabetic macular atrophy, CMT may be reduced and associated with worse BCVA, confounding this correlation.^15^ A single large intraretinal cyst in the absence of other imaging biomarkers may retain a normal BCVA compared with a DME pattern with numerous smaller-sized intraretinal cysts. In our study, greater intraretinal cyst size was associated with worse BCVA at 1 and 6 months; however, this correlation may be confounded by large macrocysts and cystoid degeneration, which confers poorer visual and morphologic outcomes.^15,23^

In conclusion, this post-hoc analysis of the DiMECAT trial shows that the microscopic features of DME can be stabilized in most patients with the appropriate use of either adjuvant IVT triamcinolone or bevacizumab during the first 6 postoperative months. The use of an OCT grading score for DME, such as the TCED-HFV, along with the presence of DRIL and disruption or absence of the EZ/ELM, may prove to be a useful tool in helping determine the visual prognosis of patients with DME who require cataract surgery.

## Supplemental Material

sj-docx-1-vrd-10.1177_24741264261423317 – Supplemental material for Biomarkers of Diabetic Macular Edema on Optical Coherence Tomography After Cataract Surgery Supplemental material, sj-docx-1-vrd-10.1177_24741264261423317 for Biomarkers of Diabetic Macular Edema on Optical Coherence Tomography After Cataract Surgery by Tuan Tran, Jonathon Goh, Sophie Rogers, Salmaan Qureshi and Lyndell L. Lim in Journal of VitreoRetinal Diseases
