# Supplementary material for: Biomarkers of Diabetic Macular Edema on Optical Coherence Tomography After Cataract Surgery
Source: J Vitreoretin Dis. 2026 Mar 19:24741264261423317. Online ahead of print. doi: 10.1177/24741264261423317 (PMC13004711; doi:10.1177/24741264261423317)
Supplement: sj-docx-1-vrd-10.1177_24741264261423317 – Supplemental material for Biomarkers of Diabetic Macular Edema on Optical Coherence Tomography After Cataract Surgery [file sj-docx-1-vrd-10.1177_24741264261423317.docx]

**Supplementary Table 1. Intergrader reliability for each OCT feature between two graders regardless of the followup timepoint at which the image was taken.**

| **OCT feature** | **No. of images graded by each grader** | **Agreement %** | **Level of agreement*** | **Unweighted Cohen’s *kappa*** |
| --- | --- | --- | --- | --- |
| Intraretinal cyst | 186 | 82.8% | Moderate | 0.731 |
| Ellipsoid zone integrity | 186 | 80.7% | Moderate | 0.680 |
| Disorganisation of the retinal inner layers | 185 | 80.5% | Moderate | 0.612 |
| Hyper-reflective foci | 185 | 91.4% | Minimal | 0.345 |
| Subfoveal fluid | 186 | 98.9% | Strong | 0.894 |
| Foveal contour | 187 | 84.0% | Moderate | 0.689 |

* level of agreement categories applied to unweighted Cohen’s *kappa* according to definitions put forward by McHugh (2012)[17] whereby, in a healthcare setting, a *kappa* value of 0.21-0.39 is “minimal agreement”, *kappa* of 0.40-0.59 is “weak agreement”, *kappa* of 0.60-0.79 is “moderate agreement”, *kappa* 0.80-0.90 is “strong agreement” and *kappa* >0.90 is “almost perfect agreement”.
